# Thoracic Spinal Arachnoid Web With Syringomyelia: A Case Report

**DOI:** 10.7759/cureus.110652

**Published:** 2026-06-11

**Authors:** Shane Weatherford, Jayanth Kumar, Katherine Roberts, Dunbar Alcindor

**Affiliations:** 1 Orthopaedic Surgery and Rehabilitation, University of Florida College of Medicine – Jacksonville, Jacksonville, USA; 2 Neurosurgery, University of Florida College of Medicine – Jacksonville, Jacksonville, USA

**Keywords:** arachnoid membrane, case report, intradural arachnoid web excision, intradural arachnoid webs, neurosurgery

## Abstract

Spinal arachnoid webs are rare intradural extramedullary lesions that most commonly occur in the thoracic spine and may cause focal dorsal spinal cord compression, cerebrospinal fluid flow obstruction, syringomyelia, and progressive myelopathy. Diagnosis can be delayed because symptoms are often nonspecific and the arachnoid web itself may be difficult to directly visualize on standard magnetic resonance imaging. The characteristic “scalpel sign,” defined by focal dorsal indentation of the spinal cord, is a key imaging feature. We present a case of a 47-year-old male patient with progressive mid-thoracic pain, left thoracic sensory disturbance, and bilateral foot numbness. Magnetic resonance imaging demonstrated a thoracic syrinx and focal dorsal indentation of the spinal cord at T8-T9 consistent with the scalpel sign. High-resolution constructive interference in steady-state imaging demonstrated a dorsal intradural arachnoid band, supporting the diagnosis of thoracic spinal arachnoid web. The patient underwent T8-T9 laminectomy, durotomy, and microsurgical excision of the arachnoid web. Postoperatively, he experienced resolution of sensory symptoms within two weeks, and follow-up imaging demonstrated interval reduction of syringomyelia. This case highlights the importance of recognizing thoracic arachnoid webs as an underdiagnosed cause of myelopathy and syringomyelia. Early recognition of characteristic imaging findings and definitive microsurgical treatment can result in favorable clinical and radiographic outcomes.

## Introduction

Spinal arachnoid webs are rare intradural extramedullary bands of arachnoid tissue that most commonly occur along the dorsal thoracic spinal cord. These lesions can produce focal dorsal compression of the spinal cord, disrupt cerebrospinal fluid (CSF) flow, and lead to syringomyelia or progressive myelopathy [1,2]. The “scalpel sign,” defined by focal dorsal indentation of the spinal cord on sagittal imaging, is the characteristic imaging feature associated with dorsal thoracic arachnoid webs [1].

Clinical symptoms may include thoracic pain, sensory disturbance, radicular-type symptoms, gait imbalance, myelopathic signs, or symptoms related to syringomyelia. Proposed etiologies include congenital arachnoid malformation, inflammation, prior trauma, arachnoiditis, or idiopathic arachnoid adhesion, although the underlying cause is often unclear [2,3]. Diagnosis may be delayed because symptoms are often nonspecific and the web itself may be difficult to directly visualize on conventional magnetic resonance imaging (MRI). Advanced imaging techniques, including high-resolution T2-weighted sequences, constructive interference in steady state (CISS) imaging, and cine MRI, may improve diagnostic confidence by demonstrating subtle intradural bands or CSF flow obstruction [2,3].

The differential diagnosis includes arachnoid cyst, idiopathic spinal cord herniation, arachnoiditis, and intradural extramedullary tumor [4]. Management ranges from observation in minimally symptomatic patients to surgical decompression for progressive symptoms, myelopathy, or syringomyelia [2,5,6]. This report presents a case of thoracic spinal arachnoid web with syringomyelia treated with T8-T9 laminectomy, durotomy, and microsurgical excision.

This work was accepted for poster presentation at the University of Florida College of Medicine - Jacksonville Celebration of Education and Research Day, June 11, 2026.

## Case presentation

A 47-year-old male patient presented with a one-year history of progressive mid-thoracic burning pain with a tight, constrictive quality. The pain was constant, rated 7/10 in severity, and worsened with movement, particularly flexion and lifting. His symptoms were associated with left-sided thoracic numbness radiating from the axilla to the anterior chest, diffuse numbness involving both feet, and mild gait instability. He denied bowel or bladder dysfunction and denied significant weakness. Prior conservative treatment included physical therapy, pain management, multimodal medications, medical marijuana, and lumbar injections without meaningful improvement.

On examination, the patient was awake, alert, and in no acute distress. Cranial nerves were grossly intact. Motor strength was 5/5 in all major muscle groups. He had 3+ hyperreflexia without clonus, a negative Hoffmann sign, and a negative Babinski sign. Gait was antalgic with mild instability. Sensory examination demonstrated decreased sensation over the left anterolateral thoracic region extending toward the axilla, consistent with a thoracic dermatomal sensory disturbance rather than a clearly isolated single-root distribution. Otherwise, sensation was grossly intact to light touch.

MRI of the thoracic spine demonstrated a syrinx extending from the cervical region to approximately T9-T10 and a focal dorsal indentation of the spinal cord at T8-T9, consistent with the scalpel sign (Figure 1). Axial imaging demonstrated dorsal flattening of the spinal cord without a discrete intradural mass or cystic lesion. Additional cervical and thoracic MRI demonstrated syringomyelia associated with the thoracic arachnoid web (Figure 2). High-resolution CISS imaging demonstrated a thin dorsal intradural band extending from the dorsal thecal sac toward the spinal cord, supporting the diagnosis of thoracic spinal arachnoid web (Figure 3).

**Figure 1:**
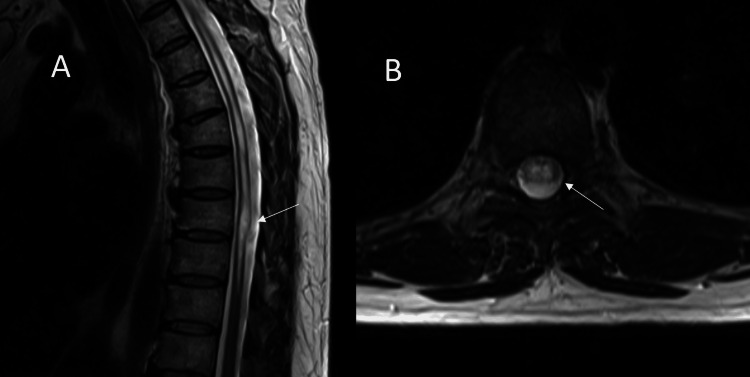
Thoracic Spinal Arachnoid Web Demonstrating the Scalpel Sign Sagittal and axial T2-weighted MRI demonstrating focal dorsal indentation of the spinal cord at T8-T9, consistent with the scalpel sign. Axial imaging demonstrates dorsal cord flattening without a discrete intradural mass or cystic lesion.

**Figure 2:**
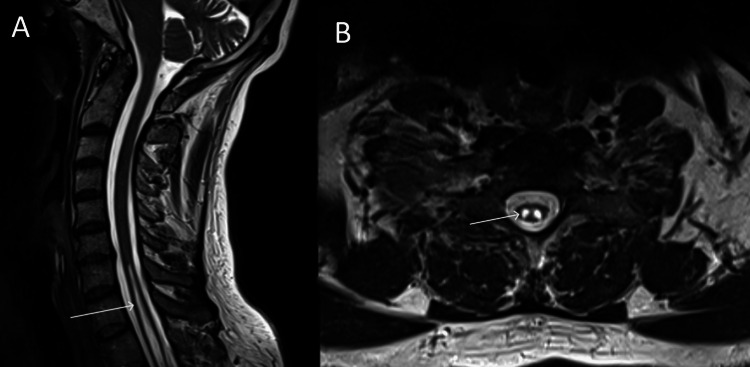
Syringomyelia Associated With Thoracic Spinal Arachnoid Web Sagittal and axial T2-weighted MRI demonstrating cervicothoracic syringomyelia associated with thoracic spinal arachnoid web.

**Figure 3:**
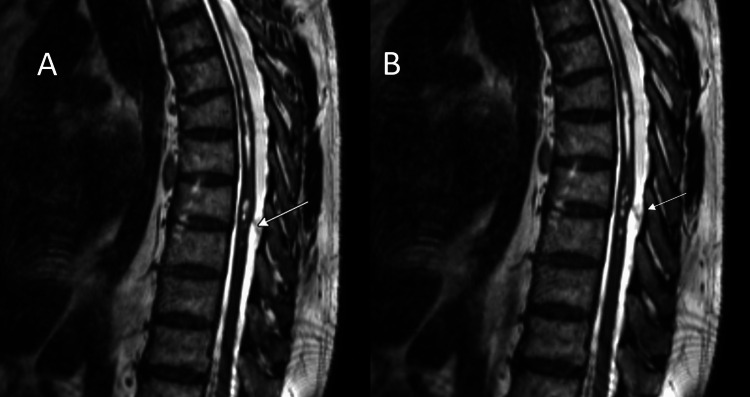
Dorsal Intradural Arachnoid Band Sagittal constructive interference in steady state (CISS) MRI demonstrating a thin dorsal intradural arachnoid band at the T8-T9 level, supporting the diagnosis of thoracic spinal arachnoid web.

Given the patient’s progressive symptoms, gait instability, syringomyelia, and imaging findings consistent with an arachnoid web at T8-T9, surgical treatment was recommended. The patient underwent T8-T9 laminectomy with midline durotomy and intradural microsurgical excision of the arachnoid web. Intraoperative fluoroscopy was used to localize the T8-T9 level, and neuroradiology assisted with level confirmation. Intraoperative neuromonitoring included upper and lower extremity somatosensory evoked potentials, upper and lower extremity transcranial motor evoked potentials, thoracic and lumbar free-run electromyography, and train-of-four monitoring.

After laminectomy and dural opening, the arachnoid was noted to be thickened. A dorsal arachnoid band compressing the spinal cord was identified. Using a microsurgical technique, the band was dissected free and excised while preserving vessels along the spinal cord and minimizing manipulation of the cord. During resection, bilateral lower extremity somatosensory evoked potentials demonstrated transient amplitude loss and latency change. Motor evoked potentials remained stable, and electromyography remained quiet. Lower extremity somatosensory evoked potentials demonstrated near-complete recovery by closure. No intraoperative complications occurred, estimated blood loss was 50 mL, and no drain was placed (Video 1).

**Video 1:** Microsurgical Excision of Thoracic Spinal Arachnoid Web Intraoperative video demonstrating microsurgical excision of a thoracic spinal arachnoid web following T8-T9 laminectomy and durotomy. The thickened dorsal arachnoid band is identified, dissected free, and excised, resulting in decompression of the spinal cord and restoration of the dorsal cerebrospinal fluid space.

Postoperatively, the patient experienced complete resolution of left-sided thoracic numbness and bilateral foot numbness within two weeks. Follow-up MRI obtained 10 months after surgery demonstrated decompression of the spinal cord and interval reduction of syringomyelia (Figure 4). At the one-year follow-up, the patient remained neurologically intact without recurrence of symptoms.

**Figure 4:**
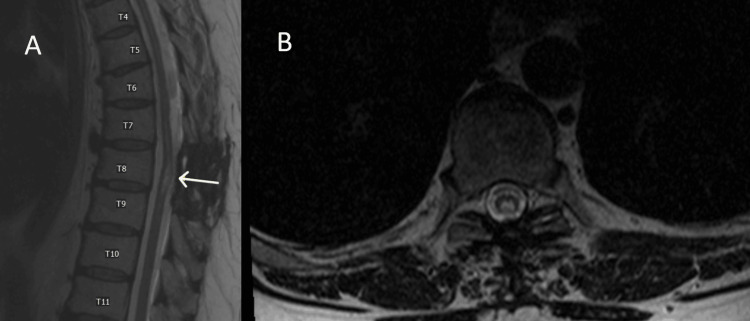
Postoperative Reduction of Syringomyelia Postoperative sagittal and axial MRI demonstrating decompression of the spinal cord and interval reduction of syringomyelia following microsurgical excision of the thoracic spinal arachnoid web.

## Discussion

Thoracic spinal arachnoid webs are rare lesions that can produce clinically significant spinal cord compression and CSF flow disturbance. Although the true incidence is unknown, published series and systematic reviews suggest that these lesions are likely underrecognized rather than exceedingly rare [2,3,5,6]. Common symptoms include axial thoracic pain, sensory disturbance, paresthesias, gait imbalance, hyperreflexia, myelopathic signs, weakness, and symptoms related to syringomyelia [1,2,5]. Less common or nonspecific presentations may include isolated neuropathic pain, radicular-type chest wall symptoms, vague lower extremity numbness, or symptoms initially attributed to degenerative spine disease [7,8]. The variability in presentation often contributes to delayed diagnosis.

The pathophysiology of arachnoid web formation remains incompletely understood. Proposed mechanisms include congenital arachnoid malformation, focal arachnoiditis, prior trauma, prior infection, inflammation, or collapse of a previously existing arachnoid cyst [2,3,7,8]. Regardless of etiology, the web acts as a focal obstruction to normal CSF flow in the dorsal subarachnoid space. This may create abnormal pressure gradients across the spinal cord, producing dorsal cord indentation and contributing to syrinx formation [1,2,8].

Imaging is central to diagnosis. The scalpel sign is highly suggestive of a dorsal thoracic arachnoid web and is characterized by focal dorsal indentation of the spinal cord on sagittal MRI [1,7]. However, the web itself is frequently too thin to be directly visualized on standard sequences. In the present case, CISS imaging helped demonstrate the dorsal intradural arachnoid band, strengthening diagnostic confidence. Advanced imaging techniques such as CISS and cine MRI may be particularly useful when conventional MRI demonstrates indirect findings but does not clearly show the web itself [3,9].

Differentiating arachnoid web from idiopathic spinal cord herniation and arachnoid cyst is important because management differs. Idiopathic spinal cord herniation typically produces ventral displacement of the cord through a dural defect, whereas arachnoid webs classically produce dorsal indentation without a ventral dural defect [4]. Arachnoid cysts may produce mass effect but are usually associated with a more defined cystic structure. In contrast, arachnoid webs often create a focal deformity without a discrete mass, making recognition of indirect imaging signs essential [1,4].

Management options described in the literature include observation with serial imaging for minimally symptomatic patients, particularly when there is no progressive neurologic deficit, and surgical decompression for patients with progressive pain, myelopathy, gait disturbance, or syringomyelia [2,5,6,10]. Surgical treatment typically involves laminectomy or laminoplasty, durotomy, intradural exploration, and microsurgical fenestration or excision of the arachnoid web. The operative goal is restoration of dorsal CSF flow while avoiding injury to the spinal cord and associated vascular structures. Reported surgical outcomes are generally favorable, with multiple series describing improvement in pain, sensory symptoms, myelopathy, and radiographic syringomyelia after decompression [2,5,6,10]. In the present case, operative excision was selected because of progressive symptoms, gait instability, syringomyelia, and imaging confirmation of a T8-T9 arachnoid web.

This case is educational because it demonstrates the full diagnostic and treatment pathway for a symptomatic thoracic arachnoid web: nonspecific thoracic pain and sensory symptoms, upper motor neuron findings, syringomyelia, the scalpel sign, confirmation with CISS imaging, intraoperative localization with fluoroscopy, neuromonitoring, and microsurgical excision. Compared with prior reports, this case reinforces the value of advanced MRI sequences when conventional imaging suggests but does not clearly define the web. The patient’s improvement in sensory symptoms and postoperative reduction in syringomyelia support restoration of CSF dynamics after decompression, although conclusions are limited by the single-case design.

## Conclusions

Thoracic spinal arachnoid webs are uncommon but clinically important causes of thoracic myelopathy and syringomyelia. In this case, the combination of progressive thoracic pain, sensory disturbance, gait instability, syringomyelia, and the scalpel sign prompted further evaluation with CISS imaging, which helped identify a dorsal intradural arachnoid band.

Prior reports describe observation for minimally symptomatic patients and surgical decompression for patients with progressive symptoms, myelopathy, or syringomyelia. In the present case, microsurgical excision was selected because of progressive symptoms and concordant imaging findings. The patient experienced clinical improvement and postoperative reduction of syringomyelia, supporting the role of operative decompression in carefully selected symptomatic cases. Larger studies are needed in the future.
